# Grand Challenges in Global Health: The Ethical, Social and Cultural Program

**DOI:** 10.1371/journal.pmed.0040265

**Published:** 2007-09-11

**Authors:** Peter A Singer, Andrew D Taylor, Abdallah S Daar, Ross E. G Upshur, Jerome A Singh, James V Lavery

## Abstract

The Grand Challenges initiative has 44 projects worldwide aimed at addressing diseases of the poor. What are the ethical, social, and cultural issues that the initiative faces?

The Grand Challenges in Global Health (GCGH) initiative is a major effort to achieve scientific breakthroughs against diseases that kill millions of people each year in the world's poorest countries. With 44 projects, more than US$450 million in funding, and scientists from 33 countries, it has the potential to greatly reduce the suffering and death that disproportionately affect the 2 billion poorest people on earth. The 14 Grand Challenges serve seven long-term goals in global health [1], which are shown in Box 1.

**Box 1. Grand Challenges in Global Health**

**Goal 1. Improve Childhood Vaccines**

Grand Challenge #1: Create Effective Single-Dose Vaccines

Grand Challenge #2: Prepare Vaccines that Do Not Require Refrigeration

Grand Challenge #3: Develop Needle-Free Vaccine Delivery Systems

**Goal 2. Create New Vaccines**

Grand Challenge #4: Devise Testing Systems for New Vaccines

Grand Challenge #5: Design Antigens for Protective Immunity

Grand Challenge #6: Learn about Immunological Responses

**Goal 3. Control Insects that Transmit Agents of Disease**

Grand Challenge #7: Develop a Genetic Strategy to Control Insects

Grand Challenge #8: Develop a Chemical Strategy to Control Insects

**Goal 4. Improve Nutrition to Promote Health**

Grand Challenge #9: Create a Nutrient-Rich Staple Plant Species

**Goal 5. Improve Drug Treatment of Infectious Diseases**

Grand Challenge #10: Find Drugs and Delivery Systems that Limit Drug Resistance

**Goal 6. Cure Latent and Chronic Infection**

Grand Challenge #11: Create Therapies that Can Cure Latent Infection

Grand Challenge #12: Create Immunological Methods to Cure Latent Infection

**Goal 7. Measure Health Status Accurately and Economically in Developing Countries**

Grand Challenge #13: Develop Technologies to Assess Population Health

Grand Challenge #14: Develop Versatile Diagnostic Tools

Such a significant investment in scientific research must be accompanied by a program addressing the ethical, social, and cultural (ESC) issues that may arise—either in the development and implementation of the research projects themselves, or in the subsequent appropriate use of resultant knowledge and technologies by communities in need (see next paper in this series by Berndtson et al. [2]).

Examples of setbacks as a result of ESC issues involving new technologies or approaches include: (1) the halting of trials of tenofovir (an antiviral medication used to treat HIV) in Cambodia, Cameroon, and Nigeria following claims there was not proper consultation with affected communities [3]; (2) rejection of white anti-malaria bed nets in cultures where that color was culturally sensitive [4]; and (3) Zambia's rejection of genetically modified foods because of perceived health, environmental, and economic risks [5].

The Bill & Melinda Gates Foundation has recognized the need to understand and address ESC issues in the GCGH initiative. In November 2005, the foundation awarded us a grant to develop and run the ESC program for the GCGH. The two goals of the ESC program are (1) to provide an advisory service for GCGH projects on ethical, social, and cultural issues in the short- to mid-term, and (2) to create a research program to facilitate appropriate adoption of the technologies resulting from the GCGH projects in the long-term. Our starting assumptions for the ESC program are that science and technology play a key role in global health and development [6], and that the appropriate development and adoption of technology requires careful attention to the salient ethical, social, and cultural issues.

In this article we describe the ESC program of the GCGH initiative. We emphasize the features of our program that we believe represent valuable innovations in the evolution of ethical and social programs in large-scale science. We also introduce three subsequent articles appearing in this issue of *PLoS Medicine* that begin to illustrate some of the contributions of the ESC program to date [2,7,8].

## What Is Already Known

To our knowledge, the first large-scale science project to address ESC issues systematically was the Ethical, Legal and Social Implications (ELSI) program of the Human Genome Project. In 1988, James Watson determined that 3% of the project's budget should be devoted to ELSI issues, raising this number to 5% in 1991 [9]. Although the ELSI program has stimulated efforts to inform the public about risks in genetic research and to deal with those concerns through research and education projects, a *Science* commentary on ELSI in 1996 revealed reservations about the program's “blurry mandate” and distance from the applications of the actual science [9]. Watson himself suggested that he saw ELSI initially as a way merely to deflect criticism from those wary of the consequences of genetic research, saying “It kept us from being attacked” [9]. An independent review of the ELSI research programs in 2000 criticized the limited number of voices informing ELSI research and recommended drawing on “theoretical perspectives from outside the traditional community of ELSI researchers” from a “broader array” of disciplines and communities [10].

The Human Genome Project's ELSI program set a standard for addressing ESC issues in large-scale science projects. Subsequent programs such as the International HapMap Project (http://www.hapmap.org/ethicalconcerns.html) and the National Nanotechnology Initiative (http://www.nano.gov/html/society/ELSI.html) have also adopted ESC programs. However, developing world involvement in these initiatives has been limited. For example, HapMap's Populations/ELSI Group, which considers ethical and sampling issues in the HapMap project, links membership to funding. Although China has managed to secure financial support, and therefore membership, Nigeria does not have the financial means to join the group, whose other members include the United States, United Kingdom, Canada, and Japan [11]. All of the participants and contributors attending the 2003 National Nanotechnology Initiative Workshop on societal implications of nanotechnology represented industrialized nations [12].

Our program aims to improve on previous programs by more successfully integrating the ESC activities with the scientific work of the GCGH investigators, and by focusing on ESC obstacles to the successful implementation of effective technologies for and with the developing world.

## Preliminary Activities: Advisory Service

The goal of the advisory service is to facilitate the successful and appropriate achievement of milestones in the GCGH projects in the short- and mid-term. Below we discuss the advisory service consultation model and give examples of two advisory service consults.

**Figure 1:**
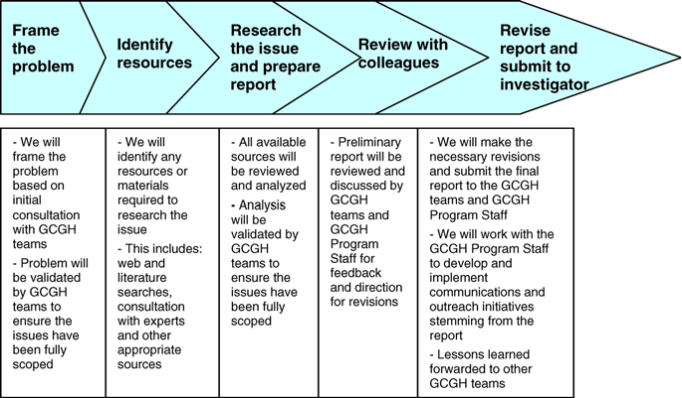
Consultation Model The design of the ESC advisory service draws from a consultation model we have successfully used in the past.

The ESC advisory service (Figure 1) is designed to address ESC issues identified at the outset of GCGH research projects, as well as challenges encountered as projects progress. At the GCGH kickoff meeting in Seattle in November 2005, we introduced the ESC advisory service to the project staff and to the GCGH program officers. We explained that our goal was not to regulate research or to provide ethical cover. We recognized the expertise and accountability of the investigators and so did not see ourselves as providing a simple guidance function. Rather, our aim was to work in a close, integrated fashion with the investigators on ESC issues arising in their projects and thus contribute directly to the achievement of their milestones.

In the first year of operation, we consulted with 24 of the 44 GCGH projects in varying levels of depth, from the achievement of specific regulatory and oversight milestones to preliminary discussions to scope out approaches to anticipated issues. The remaining 20 projects have indicated no ESC advisory needs at this time, though we will continue to engage the project's researchers to ensure any emerging ESC needs are met. Two in-depth consults (Boxes 2 and 3) illustrate the nature and scope of the advisory service and show responses and/or recommendations, as well as successes and lessons learned.

**Box 2. Genetic Strategies for Control of Dengue Virus Transmission**

*Goal 3: Control Insects that Transmit Agents of Disease*

*Grand Challenge #7: Develop a Genetic Strategy to Control Insects*

**Nature and Scope of the Advisory Service Issue**

Our first in-depth consult started with a request for guidance in the selection of a field site for caged field trials of genetically modified mosquitoes in four candidate countries: Thailand, Peru, Trinidad and Tobago, and Mexico.

**Response/Recommendation Provided**

In February 2006, the ESC advisory service conducted a site visit to Mexico to assess community and regulatory capacity for long-term engagement for caged field trials of genetically modified mosquitoes. This was followed in March 2006 with a site visit to Trinidad for a similar assessment. We then worked closely with the investigators to guide the development of a framework of ESC considerations to facilitate the site selection decision. When Tapachula, Mexico was selected as the site of the caged field trials, we began to work closely with the investigators and Mexican collaborators to develop the community engagement protocol for the project.

**Successes and Lessons Learned**

The project investigators used the ESC site selection framework to guide their final decision. The framework ensured that the decision-making process was transparent, and involved a fully developed set of criteria and rationales. By their openness and highly collaborative approach to this aspect of their project, the project investigators also helped us demonstrate the value of deep integration of ESC issues with science. A description of the site selection experience has now been submitted for publication, and we hope it will prove useful for other researchers facing similar challenges, both within and beyond the GCGH.

**Box 3. Development of Novel Mouse Models for HIV and Hepatitis C Infection**

*Goal 2: Create New Vaccines*

*Grand Challenge #4: Devise Reliable Testing Systems for New Vaccines*

**Nature and Scope of the Advisory Service Issue**

Focusing on human embryonic stem cell research in China, the challenge was to help the project meet a specific regulatory and oversight milestone. We did this by helping the team to establish an effective oversight mechanism for the project, and to help ensure that the project's standard operating procedures and guidelines would be consistent with emerging standards in the United States, China, and elsewhere. The project required the development of a high-level regulatory ethics board (REB) and an embryonic stem cell research oversight committee to provide monitoring and ethical guidance.

**Response/Recommendation Provided**

The REB milestone of this project required four main outcomes:

1. The establishment of the REB, with membership including high-level representation of US and Chinese experts.
2. The development and adoption of a decision-making charter for the REB.
3. The development and adoption of standard operating procedures related to ethical and regulatory aspects of the project.
4. A customized training program on the operating procedures.

All of these requirements have now been met. The ESC advisory service served as an expert broker, facilitating the memberships of Prof. Jonathan Moreno, University of Pennsylvania (and member, Human Embryonic Stem Cell Research Advisory Committee, US National Academies) and Prof. Jeanne Loring, Burnham Institute, from the United States, and Prof. Xiaomei Zhai of Peking Union Medical College from China, on the REB and the oversight committee.

We also played an active role in the initial drafting and development of the REB decision-making charter, the main governance instrument for the REB. Standard operating procedures were developed to provide guidance to the researchers on the project. We participated in the first training program for project research staff on regulatory and ethics issues, conducted in Beijing in July 2006.

**Successes and Lessons Learned**

This consult involved extensive deliberations and document drafting in collaboration with the US and Chinese experts, the project team, and the Bill & Melinda Gates Foundation program officer. Key lessons learned were the importance of communication and relationship building for the establishment of trust and collaboration throughout the consult, and the importance of seeking outside expertise to supplement the advisory service. We expect this experience to be extremely valuable for future GCGH consults.

Based on our experience with the first year of consults, we have implemented a plan to assign an advisory service co-leader (Jerome Singh or James Lavery) and a lead bioethicist (Paulina Tindana or Anant Bhan) to each GCGH project. In this way, ESC team members are developing Grand Challenge–specific expertise. To the extent possible, the assignment of the lead bioethicists reflects their areas of training and expertise. During the initial phase of communication, the co-leader and lead bioethicist define the issues and develop a preliminary strategy for addressing the consult.

In an attempt to stay ahead of emerging issues and consolidate our expertise for anticipated consults, we have also initiated a process of consultation with the GCGH program officers. These relationships with the program officers, who have extensive experience in the GCGH scientific areas, have been instrumental in helping us identify issues arising across Grand Challenges or within individual projects. In addition to occasionally inviting individual program officers to join our bi-weekly advisory service conference calls, we have regular participation of program officers in our bi-weekly full ESC team meetings, as well as occasional face-to-face meetings to discuss specific issues. These close working relationships have allowed us to benefit from the expertise and insights of the program officers in terms of understanding all of the relevant scientific dimensions of the projects, and to better appreciate and anticipate related ESC issues.

We have also developed a database of consults that documents the key issues, work plans, actions, and outcomes of each consult. This database serves as a detailed record of the advisory service activities, and is a key management tool in the evolution and evaluation of the advisory service.

## Preliminary Activities: Research Program

The goal of the research program is to fill gaps in knowledge in order to facilitate the successful and appropriate adoption of the technologies resulting from the GCGH projects by communities in need in the long-term. The research program was designed to address cross-cutting issues in the GCGH projects. It has been shaped by a review of the project proposals, the results of the focus groups of GCGH investigators and program staff, and the survey of key informants in the developing world. Perhaps the most significant question addressed by the research program is: Assuming some of the projects are successful and the technologies are developed, how will they reach the communities that need them in the developing world? Below we discuss four aspects of the research program that offer insight into that question.

### Working papers

Some of the working papers we have drafted scope ESC issues in the GCGH. During the November 2005 GCGH meeting, we held focus groups with all the GCGH principal investigators as well as the program officers for those grants, who are based at the Bill & Melinda Gates Foundation and the Foundation for the National Institutes of Health (which manages some of the GCGH projects), to seek their perspectives on the main ESC issues they expected to encounter in their GCGH projects. We then asked 70 key informants from the developing world about these and other ESC issues in the GCGH. The results of this research are detailed in the second paper in this series in *PLoS Medicine* [2].

Given the importance the GCGH investigators and developing world key informants attached to community engagement as well as the role of civil society organizations, we have also prepared conceptual papers on these topics. These were distributed as working papers to GCGH investigators and program staff at the 2nd Annual GCGH Meeting, and the final versions are published as the third and fourth articles in the series in this issue of *PLoS Medicine* [7,8]. In each article, we introduce the topic, briefly review what is known, present some preliminary findings from our program, and sketch next steps.

Another type of working paper generated through the ESC program arises from the unique and innovative nature of many of the GCGH projects in which the investigators face interesting and important ESC issues. As noted above, we provide guidance to investigators on these issues in the advisory service. However, many of these issues have not been adequately addressed in the literature. We believe our analyses and proposed solutions to important ESC challenges in the GCGH may be of use to the broader global health research community. The first two of these advisory service articles document our approach to site selection for caged field trials of genetically modified mosquitoes, and our efforts to reconcile US and Chinese stem cell research guidelines to ensure adequate oversight of a GCGH-funded project involving stem cell research in China (Boxes 2 and 3). These articles will be published independently. We anticipate that in the next five years of our program, we will produce about 20 publications of this type.

### Working groups

Our research has highlighted the importance of building ESC expertise on specific Grand Challenge goals, such as nutritionally enhanced foods, chemical and genetic control of insect populations, vaccines, and diagnostics. Each working group will be co-chaired by a developing world opinion leader and will include GCGH project investigators, program officers, and members of our team. The groups will develop a technology road map leading from laboratory to village. They will identify and commission research to fill current gaps in knowledge on ESC issues and will identify actions needed to ensure the successful adoption of the technologies flowing from GCGH projects.

### Global case studies to identify good practices

Over the next three to four years, we propose to conduct two global case studies: one on community engagement, and one on commercialization of health technologies in low-resource settings. The goal of these projects is to identify a set of “good practices” related to these activities for developing world settings. We hope that the insights generated by these studies will assist the GCGH investigators and the broader global health research community.

### Demonstration project on public engagement

We have developed and are beta-testing a media-rich web-based public engagement tool, WaterEngage, to function as a demonstration project meant to create a global virtual community of interest around global water problems (http://www.waterengage.com/). Among other things, the tool highlights how emerging nanotechnology and biotechnology applications can address waterborne and water-related diseases, e.g., through point of care diagnostics. We see a clear connection between the WaterEngage platform and the work of the GCGH program. We are currently developing MalariaEngage, a program which would create a virtual global community on this important topic. Ten of the 44 GCGH projects have potential relevance to malaria.

## Next Steps

We believe that the ESC program is an innovative approach to dealing with key ethical issues in a large-scale science program that seeks to improve human well-being in the developing world. Our approach combines conceptual reflection, empirical research, and service activities. It incorporates input from research programs, funders, and the broader community of potential beneficiaries of research. The key features of this program, which may serve as a model for other large-scale science initiatives, are the close linkage of ESC activities to the science projects themselves, and the extensive inclusion of voices from the developing world.

## Abbreviations

ELSI: ethical, legal and social implications
ESC: ethical, social, and cultural
GCGH: Grand Challenges in Global Health
REB: regulatory ethics board

## References

[pmed-0040265-b001] Varmus H, Klausner R, Zerhouni E, Acharya T, Daar AS (2003). Public health. Grand Challenges in Global Health. Science.

[pmed-0040265-b002] Berndtson K, Daid T, Tracy CS, Bhan A, Cohen ERM (2007). Grand Challenges in Global Health: Ethical, social, and cultural issues based on key informant perspectives. PLoS Med.

[pmed-0040265-b003] Singh JA, Mills EJ (2005). The abandoned trials of pre-exposure prophylaxis for HIV: What went wrong?. PLos Med.

[pmed-0040265-b004] World Health Organization (1997). Guidelines on the use of insecticide-treated mosquito nets for the prevention and control of malaria in Africa. http://www.who.int/malaria/docs/pushba.htm.

[pmed-0040265-b005] Schleicher A (2002 December 18). Food crisis in Zambia. NewsHour Extra. http://www.pbs.org/newshour/extra/features/july-dec02/zambia.html.

[pmed-0040265-b006] Juma C, Yee-Cheong L (2005). Innovation: Applying knowledge in development.

[pmed-0040265-b007] Tindana PO, Singh JA, Tracy CS, Upshur REG, Daar AS (2007). Grand Challenges in Global Health: Community engagement in research in developing countries. PLoS Med.

[pmed-0040265-b008] Bhan A, Singh JA, Upshur REG, Singer PA, Daar AS (2007). Grand Challenges in Global Health: Engaging civil society organizations in biomedical research in developing countries. PLoS Med.

[pmed-0040265-b009] Marshall E (1996). Genetics: The Genome Program's conscience. Science.

[pmed-0040265-b010] National Human Genome Research Institute (2000). A review and analysis of the ELSI research programs at the National Institutes of Health and the Department of Energy. http://www.genome.gov/10001727.

[pmed-0040265-b011] Foster MW (2004). Integrating ethics and science in the International HapMap Project. Nature Rev Genet.

[pmed-0040265-b012] Roco MC, Bainbridge WS (2003). Nanotechnology: Societal implications—Maximizing benefits for humanity.

